# Effects of alkali stress on antioxidant capacity, lipid metabolism, apoptosis and autophagy of *Eriocheir sinensis*

**DOI:** 10.1038/s41598-025-96808-8

**Published:** 2025-07-01

**Authors:** Jia Liu, Yihan Kou, Dexuan Kong, Zihao Yan, Zhengyao Guo, Wei Lu, Wenfa Lv, Yuehong Li

**Affiliations:** 1 College of Animal Science and Technology, College of Animal Medicine, Jilin Agricultural University, Changchun, 130118 China; 2 Ministry of Education Laboratory of Animal Production and Quality Security, Jilin Agricultural University, Changchun, 130118 China

**Keywords:** Carbonate alkalinity, *Eriocheir sinensis*, Oxidative damage, Lipid metabolism, Apoptosis, Cytokines, Lipids, Proteins

## Abstract

This study investigates the effects of alkali toxicity on Chinese mitten crab (*Eriocheir sinensis*) exposed to 17.5 mmol/L alkalinity for four weeks. Alkali toxicity resulted in significant physiological responses, notably reducing survival rates. There was a marked increase in MDA content and a decrease in SOD, CAT, and T-AOC levels in the hemolymph. Additionally, there was an elevation of ROS levels in the gills and hepatopancreas, along with *Keap1* expression, leading to tissue oxidative damage. Alkalinity exposure induced apoptosis, significantly increasing the mRNA expression of *Bax*, *caspase3*, *P53*, and a notable rise in TUNEL-positive cells. Long-term alkali exposure also reduced hepatopancreas lipid accumulation by promoting *CPT1* while inhibiting *SREBP*, *ACC*, and *CAAT* expression. Furthermore, autophagy was considerably influenced by alkali stress, as evidenced by the upregulation of mRNA expression of *ATG5*, *ATG7*, *LC3a*, *Beclin1*, as well as the protein expression of Beclin1 and parkin. Overall, the findings indicate that alkalinity exposure triggers oxidative stress, lipid metabolic disorder, apoptosis, and autophagy in *E. sinensis*, providing insights into how *E. sinensis* adapts to saline-alkali environments and sustainable development in saline-alkali waters.

**Supplementary Information:**

The online version contains supplementary material available at 10.1038/s41598-025-96808-8.

## Introduction

According to United Nations statistics, the global saline alkali land area has reached 950 million hectares, China possesses saline-alkali land area of approximately 99.1 million hectares, including 4.6 million square kilometers of low-lying saline-alkali water^1,2^. Furthermore, with the impact of climate change and human activities, including agricultural irrigation, industrial soda production, the use of deicing agents on roadways, and salt mining, the issue of salinization and alkalinization of global water resources is increasingly worsening^3^. However, the high pH and ion imbalance characteristic of saline-alkali water severely restrict the reproduction, survival, and growth of aquatic organisms. High carbonate alkalinity is a significant stress factor for aquatic animals in saline-alkali water^4^. It has been demonstrated that alkalinity stress can induce oxidative stress, immune suppression, and cell apoptosis in aquatic animals, ultimately affecting their survival and growth^5,6^. Additionally, when aquatic animals are exposed to high alkalinity water, HCO_3_^−^ and CO_3_^2−^ ions can cause damage to gill and skin, disrupt ion exchange, and affect the acid-base balance of aquatic animals, impacting their survival. Study on Pacific white shrimp (*Litopenaeus vannamei*) indicated that exceeding certain alkalinity levels results in their appendages loss of mobility, rendering them unable to respond to external stimuli, which eventually leads to death^7^.

The antioxidant defense system in aquatic creatures is markedly influenced by carbonate alkalinity, under alkali stress, antioxidant enzyme activities are inhibited, which weakens the antioxidant capabilities of aquatic animals^2,5^. Besides, carbonate alkalinity could induce excessive reactive oxygen species (ROS) production in aquatic organisms, leading to a significant accumulation of lipid peroxides and causing oxidative stress^8^. In the Chinese mitten crab (*Eriocheir sinensis*), the increased alkalinity levels (17.50 and 35.00 mmol/L) over 96 h exposure caused a significant and dose-dependent rise in ROS levels^3^. ROS play a central role in regulation of the main pathways of apoptosis mediated by mitochondria, death receptors and the endoplasmic reticulum (ER)^9^. Apoptosis, a form of regulated cell death, is crucial for maintaining normal growth and cellular balance^10^. Studies have found that acute alkalinity stress can promote hepatopancreatic apoptosis in Pacific white shrimp^11^. Apoptosis is also related to autophagy, and interference with the formation of autophagosomes or lysosome activities may affect the apoptosis process^12^. Research reports that autophagy-related 5 (ATG5) mediates the release of cytochrome c and caspase activation through interaction with members of the B-cell lymphoma 2 (Bcl2) family, promoting apoptosis^13^. Study have found that carbonate exposure induce liver autophagy and abnormal lipid metabolism in crucian carp^1^.

In decapod crustaceans, the hepatopancreas is the largest organ of the digestive gland and is the main metabolic organ, which is responsible for the absorption and metabolism of nutrients, synthesis of digestive enzymes and proteins^14^. Acylcarnitine is an intermediate product of lipid metabolism and an essential substance for cellular energy metabolism. Under saline-alkali stress, the levels of acylcarnitine in the *E. sinensis* undergo significant changes, indicating that saline-alkali stress has an impact on the lipid metabolism of the *E. sinensis*^2^. However, no studies have shown the effects of chronic alkalinity stress on hepatopancreas in the *E. sinensis.*

*E. sinensis*, known for its rich nutritional content and distinctive flavor, is a prominent freshwater crustacean in China with substantial market demand. It demonstrates notable resilience to alkaline conditions, with a 96 h median lethal concentration (LC50) of 69.74 mmol/L of alkalinity, and to ensure safety, a concentration below 18.25 mmol/L is recommended^2^. However, it is still unknown whether long-term alkalinity stress at the “safe concentration” level can cause harm to the *E. sinensis*. Additionally, fattening in saline-alkaline water can enhance both the nutritional content and taste profile of *E. sinensis*^15^. However, breeding *E. sinensis* successfully in highly alkaline waters remains challenging. Thus, this research aimed to examine the impact of long-term carbonate stress on the tissue structure, antioxidant capacity, lipid metabolism, apoptosis, and autophagy of *E. sinensis*.

## Materials and methods

### *E. sinensis* culture conditions and experimental design

Eighty healthy *E. sinensis* (30.33 ± 0.27 g) were obtained from a farm in Jilin Province. They were placed in eight glass tanks (100 × 50 × 60 cm) with a water temperature maintained at 21–22 °C, and dissolved oxygen levels of at least 7 mg/L for a two-week acclimation period. Once the experiment began, the *E. sinensis* were divided into two groups with carbonate alkalinity concentrations of 0 and 17.5 mmol/L, each group consists of four replicates, with ten *E. sinensis* per replicate, and acclimated for four weeks. These concentrations were set according to the studies based on pre-experiment (results in supplementary materials, Figs. S1, S2 and S3). Throughout the experiment, the water was changed daily and water quality related indicators are shown in Table S1. During the period of acclimatization and conducting experiments, *E. sinensis* was hand-fed three times a day, the feeding time is 8:00, 13:00 and 18:00, feed ingredients include crude protein (≥ 32.0%), crude fat (≥ 4.0%), crude fiber (≤ 6.0%), crude ash (≤ 16.0%), calcium (≥ 5.0%), total phosphorus (≥ 1.0%), lysine (≤ 1.6%), and moisture (≤ 11.0%).

### Sample collection

Upon completing the culture trial, the *E. sinensis* in each tank were counted and weighed. *E. sinensis* were anesthetized with ice water to dissection or blood sampling. The hemolymph, gills, and hepatopancreas of *E. sinensis* were collected and placed in storage tubes without enzymes and stored at -80 °C for mRNA and protein expression testing. The hemolymph was extracted from the third appendage joint of the crab using a syringe and added to an enzyme-free centrifuge tube containing an anticoagulant, and centrifuged at 4000 g for 10 min, the supernatant was collected and stored at -20 °C for subsequent biochemical analysis^16^. Additionally, samples of the gills and hepatopancreas from nine individuals were collected for histological observation, TUNEL staining, ROS analysis, and immunofluorescence. All experimental protocols in the study were performed in keeping with the NIH Guide for the Care and Use of Laboratory Animals and approved by the Institutional Animal Care and Use Committee of Jilin Agricultural University.

### Biochemical analysis

The levels of hemolymph superoxide dismutase (SOD, A001-3-2), catalase (CAT, A007-1-1), total antioxidant capacity (T-AOC, A015-2-1), and the contents of malondialdehyde (MDA, A003-1-2) were tested using kits purchased from Nanjing Jiancheng, China.

### Histological observation

Oil red staining was conducted by Servicebio, China. The hepatopancreas were fixed in fat-specific fixative solution, then making frozen sections and stained with Oil Red O. Observations were made using an optical microscope, and photographs were taken.

### ROS test

The ROS assay kit was sourced from Servicebio, China. The method for conducting the ROS fluorescence frozen section assay follows Chi et al.^17^. Frozen sections of gills and hepatopancreas were prepared, followed by the addition of ROS stain. These were incubated at 37 °C for 30 min, shielded from light. DAPI dye solution was then applied, with an additional 10 min incubation. Finally, images were captured and quantified by Image J.

### TUNEL dyeing

Tissue sections from three *E. sinensis* specimens were prepared and examined using the fluorescein (FITC) TUNEL cell apoptosis detection kit from Servicebio, China. Images of the gills and hepatopancreas tissues were captured with a fluorescence microscope (IX71, Olympus Corporation, Tokyo, Japan). TUNEL-positive cells were identified by their green fluorescence, and the fluorescence intensity was detected by using Image J.

### Immunofluorescence microscopy

Immunofluorescence was conducted to assess Keap1/Nrf2 in gills and hepatopancreas tissues, and imaging was performed by Servicebio (Wuhan, China). Paraffin sections were prepared, sealed, and treated with Keap1 and Nrf2 antibodies (Servicebio, Wuhan, China), then incubated at 4 °C overnight. Subsequently, secondary antibodies were applied (Servicebio, Alexa Fluor 488 Goat anti-Rabbit). A self-fluorescence quenching agent was introduced, and an anti-fluorescence quenching sealing agent was used to seal the sections. Finally, immunofluorescent images were captured using a fluorescence microscope.

### Real-time quantitative PCR analysis

RNA was extracted from the gills and hepatopancreas using Trizol reagent, following the methodologies of Wang and Yin^18,19^. The cDNA was synthesized using a reverse transcription kit and stored at − 80 °C for RT-qPCR analysis. The qPCR reactions, containing 1 µL cDNA (1000 ng of reverse-transcribed RNA), 10 µL SYBR Green PCR Master Mix (2×, Takara, Japan), 1 µL each specific primer, and 7 µL ddH_2_O. The PCR conditions were as follows: 94 °C for 30 s, 40 cycles of 94 °C for 3 s, 60 °C for 30 s. Each qPCR were tested in triplicate. The genes analyzed included *CAT*, *SOD*, *Keap1*, *Nrf2*, *Bax*, *Bcl-2*, *P53*, *Caspase 3*, *ATG5*, *ATG7*, *Beclin1*, *LC3a*, *FAS*, *CPT1*, *CPT2*, *SREBP*, *ACC*, and *CAAT*. The sequences of these genes are listed in Table 1 and more details are provided in Table S2. Each sample was tested in triplicate. The relative expression levels of genes were analyzed by the 2^−ΔΔCT^ method^20^.

**Table 1:** The sequences of genes.

Type	Genes	Primer sequence (5′–3′)
Internal reference	*β-actin-*F	CAGGAAATGACCACTGCCGC
	*β-actin-*R	CGGAACCTCTCATTGCCGA
Apoptosis	*Caspase3*-F	AGGAAAAGTTCACGCCGCTA
	*Caspase3*-R	GGCTGCCTTCTGTCAGGATT
	*Bcl2*-F	GCTCAGGGCAGCGTGT
	*Bcl2*-R	GCAACCCAGACTCAATCAA
	*p53*-F	ATGTGCCTTGGCTCCAGTGTTG
	*p53*-R	TCGTCAGTCTTGATGTCTCGTGTG
	*Bax*-F	AGAGATGAAGCAGACCACGC
	*Bax*-R	TTCTACGGTGGGTGAGTCCA
Antioxidant	*Keap1-*F	CAACACCTTCATTGAGCAGCAC
	*Keap1-*R	CATTGTACACGTCCTTCTCGTCT
	*SOD*-F	GATGAAGCGCGTGTGATTCGT
	*SOD*-R	TATGGCTAAACATCGCCGCA
	*CAT*-F	ATCCTGCTGCAGGACATCCAA
	*CAT*-R	TGATGTCGTGGGTGACCTCAAA
	*Nrf2*-F	GCATCCTTCTGGTACCTCGTT
	*Nrf2*-R	CACTGCTTTGGCTCATCCTTG
	*HSP70*-F	GGCAAGGCAGCGAAGGTCATC
	*HSP70*-R	CGGCATTGGTGACAGACTGACG
	*HSP90*-F	TCACCAACGACTGGGAGGAT
	*HSP90*-R	CAGGAAGAGGAGTGCCCTGA
Autophagy	*Atg5*-F	ACCAGCAGGACGCAGAGATGT
	*Atg5*-R	GTGTGAGAAGTGTGCCGTGAGG
	*Atg7*-F	TCCGACTTCATCCGAAAATACC
	*Atg7*-R	GCACTCAACCCCAAGCCTG
	*Beclin1*-F	GCCCATATACTGTGGCGAGG
	*Beclin1*-R	CCAGGTCAAAGAGCCCAGTT
	*Lc3a*-F	ACGTCACGATGGGAGAACTG
	*Lc3a*-R	GTGGTGGTGCTCGTAAACCT
Lipid metabolism	*FAS*-F	GTCCCTTCTTCTACGCCATCC
	*FAS*-R	CGCTCTCCAGGTCAATCTTCAC
	*CPT-1-F*	CATCTGGACACCCACCTCCA
	*CPT-1-R*	ATCTCCTCACCCGGCACTCT
	CPT-2-F	AGCAGGCAGTGGCTCAGTTTA
	CPT-2-F	AAGGCAAGGAAGGGGTTGTAG
	*CAAT-F*	CATCAAGAGCCAGGAGCCCA
	*CAAT-R*	CTTCAACAGCAGCCCGCAAA
	*SREBP-F*	AGGGCTTCCAGCACGAC
	*SREBP-R*	CTTTGCCACAGATAACAGACG

### Western blotting

The western blot technique was conducted following the protocol by Li et al.^21^. Total protein of gills and hepatopancreas in *E.sinensis* were extracted by protein extraction kit (Solarbio, China) and quantified by BCA kit (Solarbio, China). Protein bands were separated by 10% sodium dodecyl sulfate-polyacrylamide gel electrophoresis using SWE fast electrophoresis buffer at a constant 220 V for 30 min. The protein bands were transferred to a PVDF membrane by electrophoresis. Primary antibodies employed included Parkin, Beclin1, Bax, Bcl-2, caspase3, and β-actin (Table 2). Protein detection was performed using an enhanced chemiluminescence system, and ImageJ software was utilized for analyzing the gray values of protein bands.

**Table 2:** Manufacture information about antibodies.

Antibody	Manufacturer	Catalog no.	Dilution rate	Source	Host species specificity	Isotypes
β-actin	Abclonal	AC026	1:50000	Rabbit	Human, Mouse, Rat, Chicken, Zebrafish, pig	IgG
Beclin1	Abmart	T550925	1:1500	Rabbit	Human, Mouse, Rat	IgG
Parkin	Wanlei	WL02512	1:500	Rabbit	Human, Mouse, Rat	IgG
Bax	Abclonal	A15646	1:750	Rabbit	Human, Mouse, Rat	IgG
Bcl-2	Abclonal	A19693	1:750	Rabbit	Human, Mouse, Rat	IgG
Caspase3	Wanlei	WL01992	1:500	Rabbit	Human, Mouse, Rat, Rabbit	IgG
Goat anti-Rabbit	Abclonal	AS014	1:5000	Goat	Rabbit	IgG

### Statistical analysis

Three separate experiments at least were performed and the data were analyzed using SPSS 20.0 and presented as Mean ± S.E.M. A Student’s t-test determined the differences between groups. The survival rate of the *E. sinensis* were analyzed by Kaplan–Meier test. All data were tested for normality and homoscedasticity using the Shapiro-Wilk and Levene ’s equal variance tests, respectively. A P-value of less than 0.05 was considered indicative of a significant difference between the two groups.

## Results

### Survival rate

**Fig. 1:**
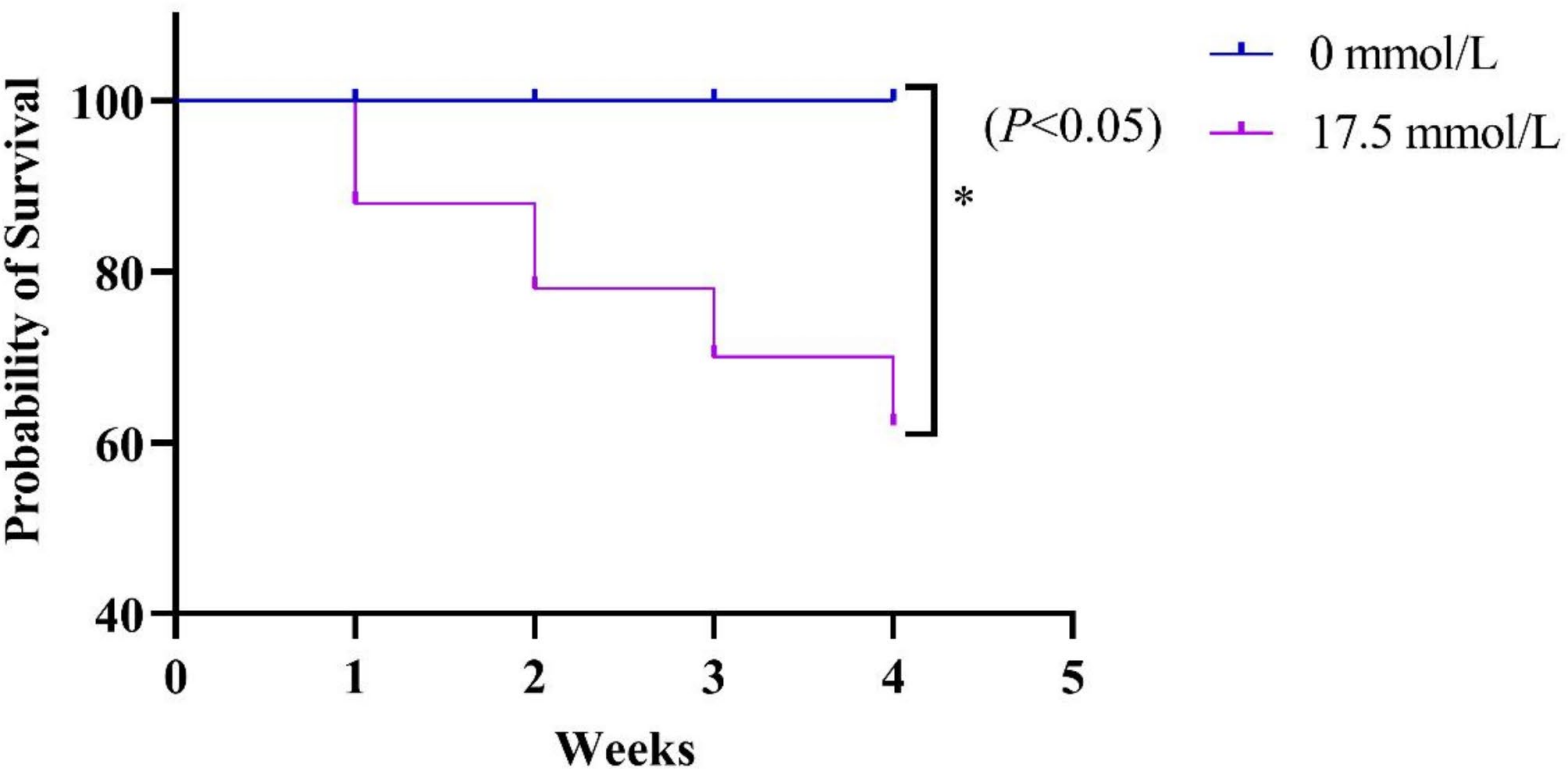
Survival rate of *Eriocheir sinensis* exposed to carbonate alkalinity. Asterisks indicate significant differences between two groups (*P* < 0.05).

The survival rate in 0 mmol/L is 100%, and in 17.5 mol/L group is 62%. Compared with 0 mmol/L carbonate alkalinity, 17.5 mmol/L carbonate alkalinity stress significantly decreased the survival rate (Fig. 1) (*P* < 0.05).

### Antioxidant capacity

**Fig. 2:**
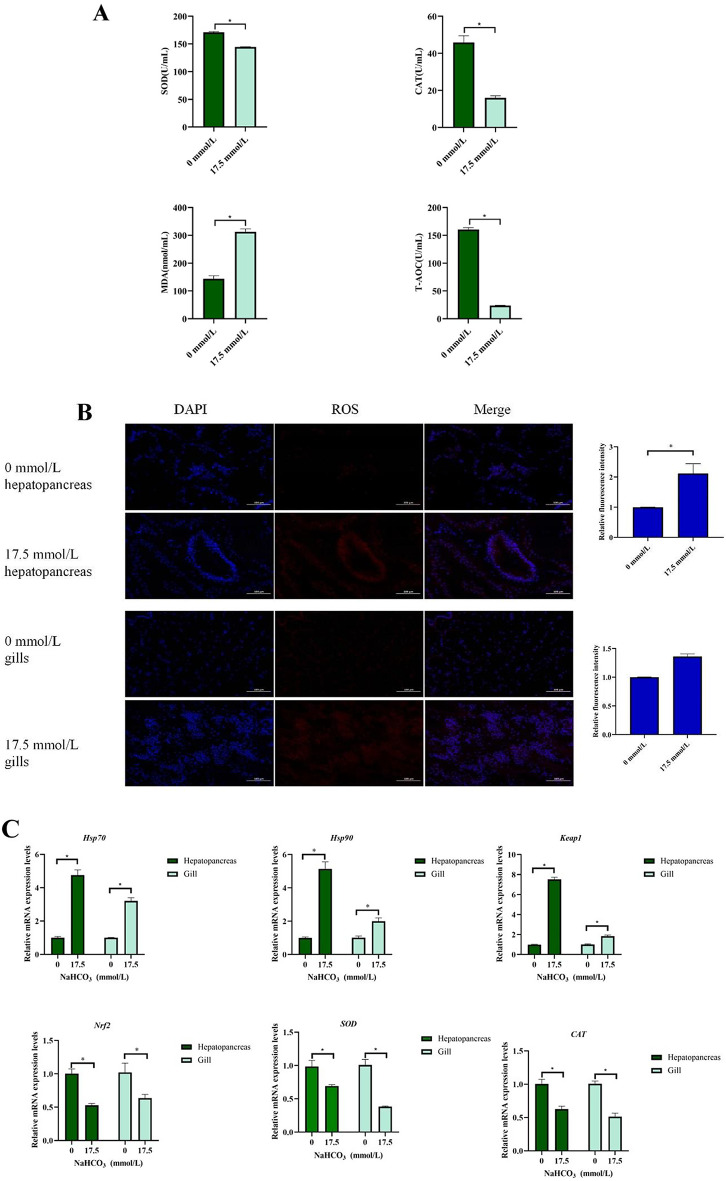

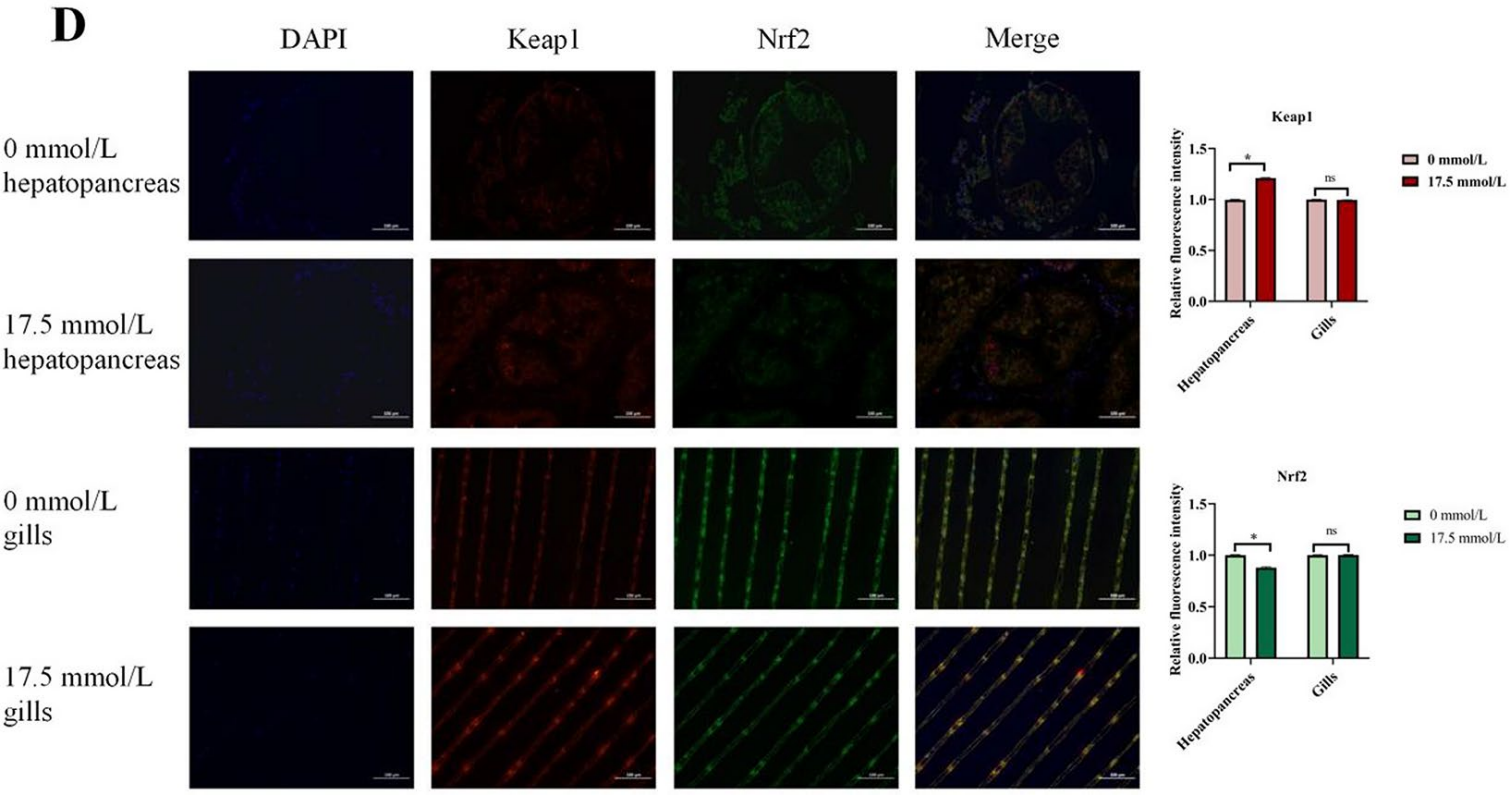
Effect of carbonate alkalinity exposure on oxidative stress of *Eriocheir sinensis*. (**A**) The influence of carbonate alkalinity exposure on oxidative stress related enzyme activity; (**B**) The influence of carbonate alkalinity exposure on reactive oxygen species levels in hepatopancreas and gills of *Eriocheir sinensis*; (**C**) The influence of carbonate alkalinity exposure on oxidative stress related genes expression; (**D**) The influence of carbonate alkalinity exposure on immunofluorescence of Keap1 and Nrf2 in hepatopancreas and gills of *Eriocheir sinensis*. Asterisks indicate significant differences between two groups (*P* < 0.05).

The activities of SOD, CAT, and T-AOC, along with the MDA content, are illustrated in Fig. 2A. Compared to carbonate alkalinity concentrations of 0 mmol/L, the activities of SOD, CAT, and T-AOC significantly decreased at 17.5 mmol/L, while MDA content significantly increased (*P* < 0.05).

The results for ROS (Fig. 2B) indicated that adding 17.5 mmol/L of carbonate alkalinity significantly increased the intensity of ROS fluorescence in the hepatopancreas (*P* < 0.05).

The mRNA expression levels of *Keap1*, *Nrf2*, *SOD*, *HSP70*, *HSP90* and *CAT* are illustrated in Fig. 2C. Compared to the control group, alkalinity stress significantly increased the mRNA expression of *Keap1*, *HSP70*, and *HSP90*, while decreasing the expression of *Nrf2*, *SOD*, and *CAT* in the gills and hepatopancreas (*P* < 0.05).

The immunofluorescence results (Fig. 2D) demonstrate that, compared to the 0 mmol/L group, 17.5 mmol/L alkalinity exposure enhanced the fluorescence intensity of Keap1, while weaken the fluorescence intensity of Nrf2 in hepatopancreas.

### Lipid metabolism

**Fig. 3:**
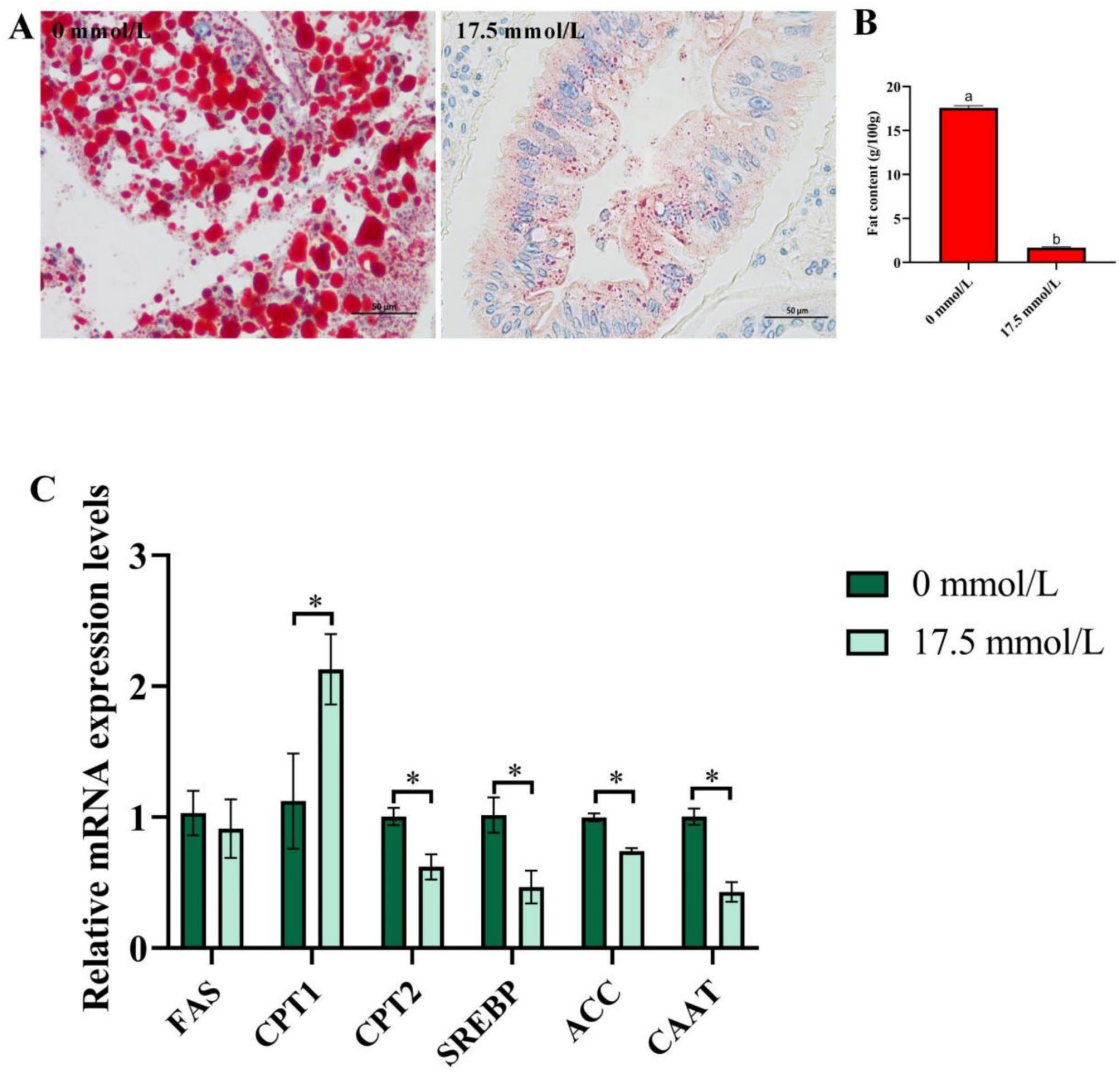
Effect of carbonate alkalinity exposure on lipid metabolism of *Eriocheir sinensis*. (**A**) The influence of carbonate alkalinity exposure on lipid accumulation of *Eriocheir sinensis*; (**B**) The influence of carbonate alkalinity exposure on fat content of *Eriocheir sinensis*; (**C**) The influence of carbonate alkalinity exposure on lipid metabolism related genes expression. Asterisks indicate significant differences between two groups (*P* < 0.05).

As illustrated in Fig. 3A, the 17.5 mmol/L group exhibited a significant reduction in the number of lipid droplets in the hepatopancreas compared to the 0 mmol/L group (*P* < 0.05). As shown in Fig. 3B, compared with 0 mmol/L group, the fat content in 17.5 mmol/L group was significantly decreased (*P* < 0.05).

The mRNA expression levels of *FAS*, *CPT1*, *CPT2*, *SREBP*, *ACC*, and *CAAT* are illustrated in Fig. 3C. Compared to the 0 mmol/L group, alkalinity stress led to an increase in *CPT1* mRNA expression, while it reduced the expression of *CPT2*, *SREBP*, *ACC*, and *CAAT* in the hepatopancreas (*P* < 0.05).

### Apoptosis analysis

**Fig. 4:**
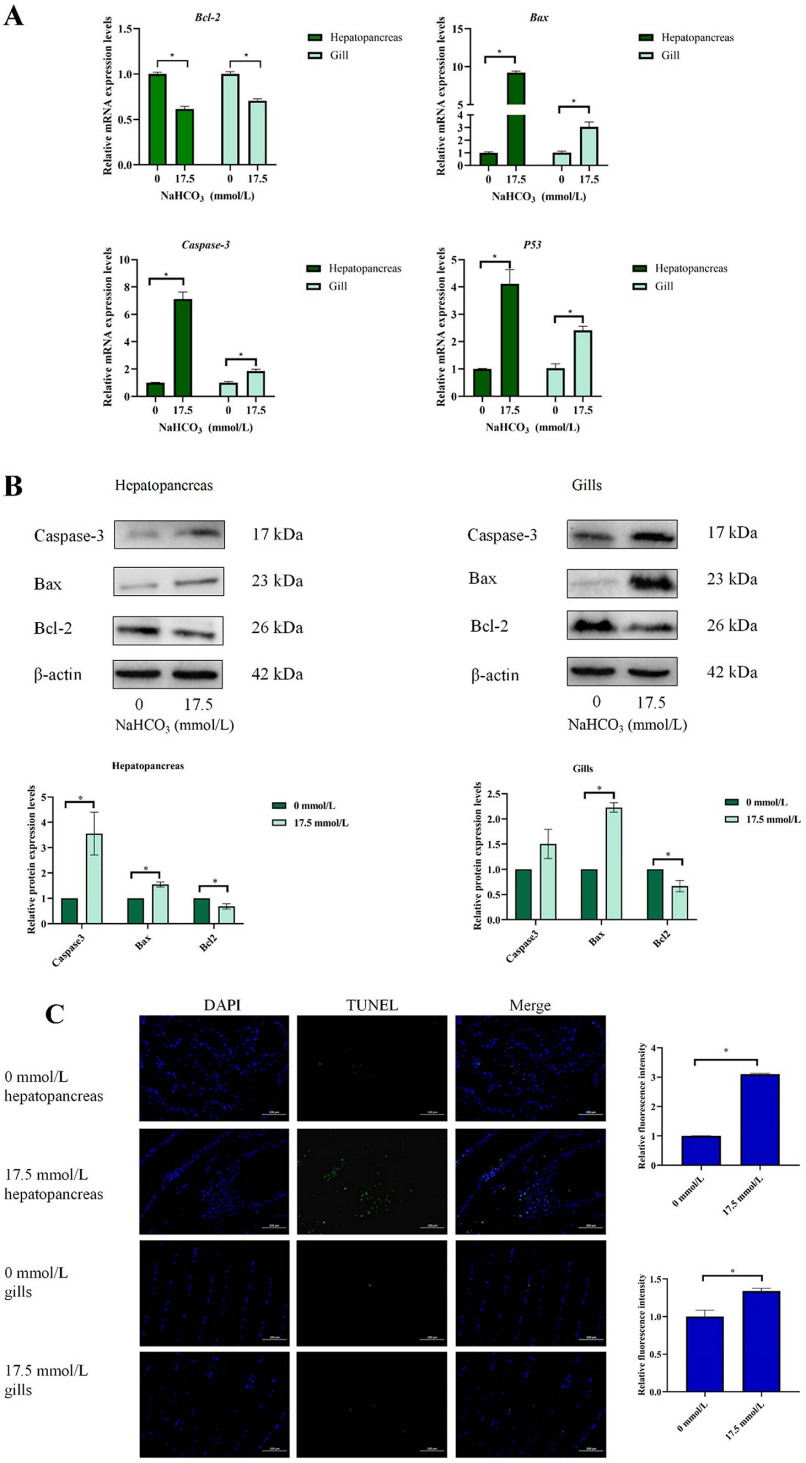
Effect of carbonate alkalinity exposure on apoptosis of *Eriocheir sinensis*. (**A**) The influence of carbonate alkalinity exposure on apoptosis related genes expression; (**B**) The influence of carbonate alkalinity exposure on apoptosis related proteins expression; (**C**) DAPI and TUNEL staining (200×). Asterisks indicate significant differences between two groups (*P* < 0.05).

The mRNA and protein expression of apoptosis-related genes and proteins are shown in Fig. 4A and B (The original image of the protein is shown in Fig. S4). Compared with the 0 mmol/L group, the 17.5 mmol/L group markedly increased the mRNA levels of *Caspase-3* and *P53* and significantly decreased the levels of *Bcl-2* in the gills and hepatopancreas (*P* < 0.05). Compared with the 0 mmol/L group, the 17.5 mmol/L group markedly increased the protein levels of Bax and significantly decreased the levels of Bcl-2 in the gills and hepatopancreas (*P* < 0.05), while caspase 3 only significantly increased in hepatopancreas (*P* < 0.05).

The TUNEL stain results are presented in Fig. 4C and Table S3. Compared to the control group, the alkalinity-treated group showed a significant increase in TUNEL levels in the hepatopancreas and gills (*P* < 0.05).

### Autophagy

**Fig. 5:**
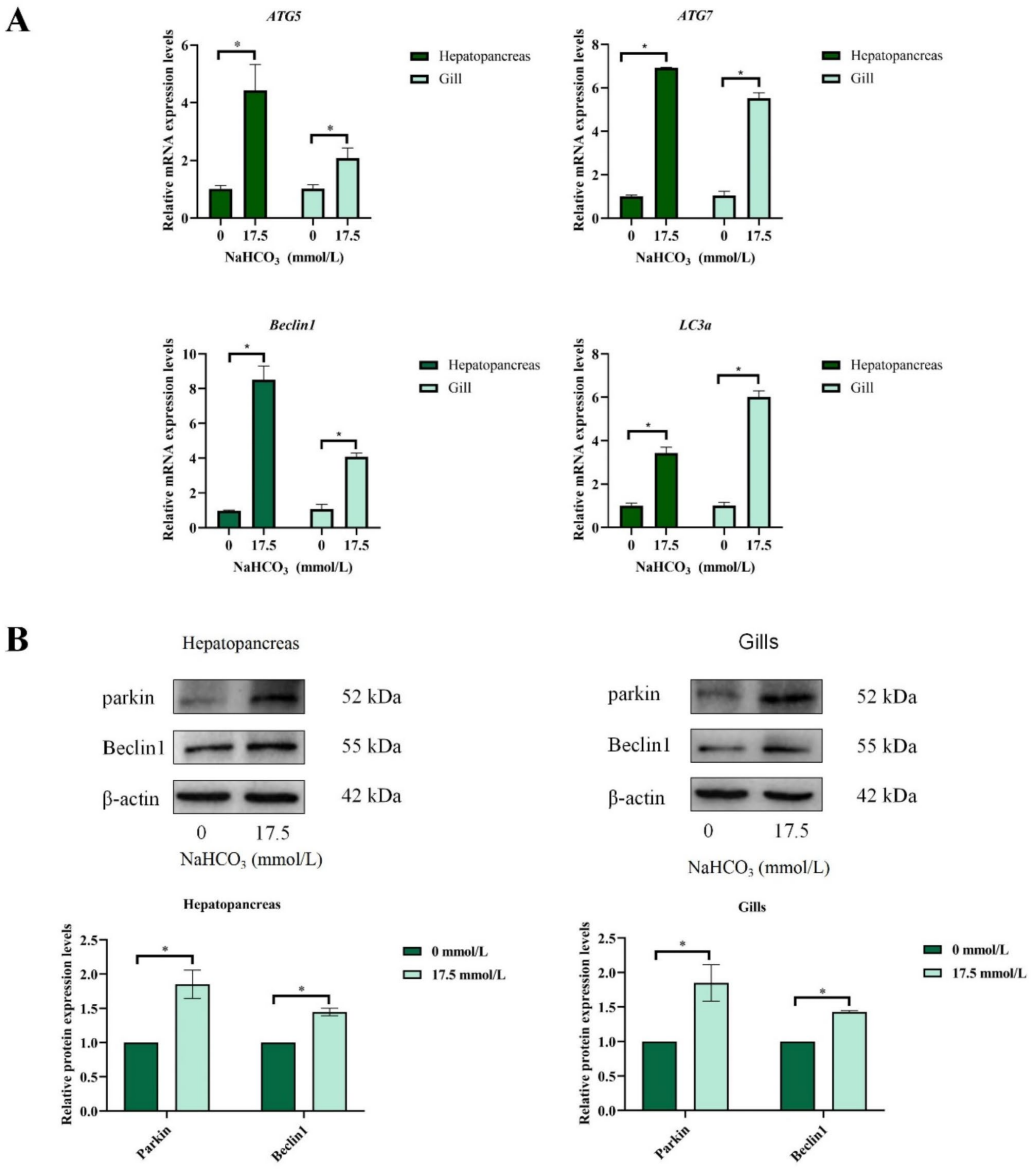
Effect of carbonate alkalinity exposure on apoptosis of *Eriocheir sinensis*. (**A**) The influence of carbonate alkalinity exposure on apoptosis related genes expression; (**B**) The influence of carbonate alkalinity exposure on apoptosis related proteins expression. Asterisks indicate significant differences between two groups (*P* < 0.05).

As shown in Fig. 5 and Table S4, the mRNA expression of *ATG5*, *ATG7*, *Beclin1* and *LC3a* in 17.5 mmol/L group was significantly higher than that in the 0 mmol/L group (*P* < 0.05). Similarly, the protein expression of Beclin1 and Parkin in 17.5 mmol/L group was significantly higher than that in the 0 mmol/L group (*P* < 0.05).

## Discussion

Carbonate alkalinity is a crucial factor affecting the normal growth and development of aquatic animals in saline-alkali waters^8^. Alkali stress can lead to a decrease in the organism’s antioxidant capacity, tissue damage, cell necrosis, and other adverse effects^22^. In this experiment, alkalinity stress reduced the survival rate, led to an increase in levels of ROS, and promoted oxidative stress of *E. sinensis*. Tao et al.^3^ have found that, after 96 h carbonate alkalinity stress, the survival rate of *E. sinensis* gradually decreased with rising alkalinity. This is similar to the findings of this study, indicating that carbonate stress affects the survival rate of *E. sinensis*. This may be because alkalinity stress induces disorders in ammonia metabolism in *E. sinensis*, leading to the accumulation of ammonia in the hemolymph, causing ammonia toxicity, and ultimately affecting its survival^3,5^. Moreover, it may also be affected by oxidative damage. Excessive ROS is detrimental to both cell growth and survival because it damages cellular components such as cell membranes and proteins^23,24^. In this study, ROS and MDA levels significantly increased, while SOD and T-AOC levels markedly decreased. The results indicate that alkalinity stress induces oxidative damage in *E. sinensis*. Additionally, we tested the mRNA expression related to antioxidant capacity. The results show that alkalinity stress significantly increased the mRNA expression of *Keap1*, *HSP70*, and *HSP90*, while significantly decreased that of *Nrf2*, *CAT*, *and SOD*. Heat shock proteins (HSPs) are involved in protection against various environmental stresses, and their expression always surges once the free radicals reach a certain level^25^. In this study, the expression of *HSP70* and *HSP90* was upregulated by *E. sinensis* to repair body cells in response to free radicals generated by saline-alkali stress. In juvenile Chinese mitten crab, the T-2 toxin suppresses the activities of T-SOD, GPx and T-AOC by inhibiting the Nrf2 signal pathway^26^. Under normal conditions, *Keap1* and *Nrf2* combine with each other to form a dimer in the cytoplasm. However, when fish are stimulated, the balance of intracellular oxidation and antioxidation is disrupted, leading to the uncoupling of *Keap1* and *Nrf2*. Nrf2 then binds to antioxidant elements such as *HO-1* to enhance cellular antioxidant capacity^27,28^. The Keap1/Nrf2 pathway is pivotal in mitigating oxidative stress, with Nrf2 playing a key role in responding to various environmental pollutants^29–32^. Similar results were found in this study, alkalinity stress decreased the expression of *Nrf2* and the activities of SOD, CAT and T-AOC, suggesting that alkalinity stress impairs the antioxidant ability and causes oxidative damage in crabs.

Nrf2 regulates cellular oxidative stress and is involved in lipid metabolism in adipocytes. Whether carbonate stress can affect lipid metabolism of *E. sinensis* by regulating the expression of *Nrf2* remains unclear. The hepatopancreas plays a crucial role in lipid metabolism in aquatic animals^33^. In this study, alkalinity stress influenced lipolysis genes (*CPT1* and *CPT2*) and lipid synthesis genes (*FAS*, *ACC*, *CAAT* and *SREBP*). CPT1 and CPT2 are closely related enzymes in the fatty acids beta-oxidation pathway, collectively participating in fatty acid oxidation^34^. In the current study, *CPT1* expression increased when *E.sinensis* was exposed to 17.5 mmol/L alkalinity, alongside a decrease in *CPT2* expression. This may be because under alkalinity stress, the *E. sinensis* might increase the expression of *CPT1* to enhance fatty acid oxidation, in order to produce more energy to cope with environmental stress. The decrease in *CPT2* may be due to changes in the internal environment of the mitochondria (such as changes in pH) or mitochondria damage leading to a reduction in its activity^35^. *SREBP* positively regulates the mRNA expression of *FAS* and *ACC*, contributing to fatty acid synthesis^36^. Nrf2 affects adipogenesis by interacting with *SREBP-1*^37^. In *E. sinensis*, SREBP has been found to positively regulate fatty acid synthesis while inhibiting fatty acid β-oxidation^38^. These findings are consistent with this study, which alkaline stress reduced the expression levels of *SREBP*, *FAS*, *ACC*, and *CAAT*. Therefore, we speculate that under alkali stress, the expression of *Nrf2* is suppressed, which in turn affects its downstream lipid metabolism-related genes, such as *CPT1* and *SREBP*, thereby promoting fatty acid breakdown and inhibiting fatty acid synthesis in *E. sinensis*.

Apoptosis, a form of programmed cell death governed by multiple genes, plays a crucial role in animal immune defense and cellular homeostasis and is linked to oxidative stress^39^. In this study, exposure to alkalinity significantly increased the number of apoptotic cells in the hepatopancreas. Additionally, alkalinity stress elevated the expression of *Bax*, *Caspase 3*, and *P53*, while it decreased the expression of *Bcl-2*. Research indicates that the balance between the pro-apoptotic protein Bax and the anti-apoptotic protein Bcl-2 is essential for regulating apoptosis^40^. Apoptosis is also influenced by the expression of *Nrf2*. In Pacific white shrimp, silencing *Nrf2* reduced antioxidant capacity and increased apoptosis^30^. Additionally, apoptosis induced by alkalinity stress may also be influenced by ROS. When excessive ROS that are not promptly removed, it can attack mitochondrial membrane lipids and proteins. This leads to the inactivation of membrane receptors and opening of the mitochondrial membrane permeability transition pore (MPTP), resulting in increased membrane permeability and reduced mitochondrial membrane potential. This subsequently activates caspases and releases apoptosis-inducing factors, eventually culminating in apoptosis. Therefore, we suspect that apoptosis induced by alkalinity stress is related to changes in ROS and *Nrf2*.

Autophagy maintains intracellular homeostasis and regulates cell growth, differentiation, and development. In addition, autophagy is involved in immune and inflammatory responses, nutrient metabolism, and resistance to microbial infection^41^. Furthermore, studies have found that autophagy is linked to lipid metabolism. Study in giant river prawn (*Macrobrachium rosenbergii*) found that 100 mg/kg tea tree oil induces autophagy to enhance lipid droplet breakdown and maintain lipid homeostasis^42^. However, either inhibition or overactivation of autophagy can lead to cellular damage^43^. Environmental stress affects autophagy and lysosomal systems, leading to the accumulation of harmful substances^44^. ROS produced by mitochondria are potent inducers of autophagy during nutrient deprivation, ischemia, and hypoxia^45^. ROS can induce autophagy by activating *p53* and *p38*, promoting the expression of ATG genes and *Beclin-1*^24^. In *E. sinensis*, copper exposure increases MDA content and upregulates the expression of *ATG7*, *Beclin1*, and *LC3a*, thereby activating autophagy^16^. In this study, alkalinity stress significantly increased the mRNA expression of *ATG5*, *ATG7*, *Beclin1* and *LC3a*. LC3 is an autophagosome marker that exists on the surface of the autophagosome and is increased during autophagosome formation^46,47^. Beclin1 is essential for autophagosome formation, shows increased expression during autophagy^48^. ATG5 has long been identified as crucial for autophagy^49^. The results indicate that alkalinity exposure enhances autophagy in the hepatopancreas and gills of crabs. This is possibly due to the oxidative stress and cellular damage caused by alkaline stress, thus activating autophagy.

## Conclusion

In this study, we investigated the detrimental impacts of alkaline exposure on the hepatopancreas and gills of *E. sinensis*. Alkaline exposure diminished antioxidative capacities and enhanced lipid peroxidation, leading to oxidative damage. Prolonged alkalinity suppressed lipid synthesis-associated genes while stimulating the expression of lipolysis-related genes, disrupting lipid metabolism. Following alkaline exposure, there was an upregulation of apoptosis-associated genes and proteins, indicating the activation of apoptosis. Furthermore, the elevated levels of autophagy-related genes and proteins in the hepatopancreas and gills suggested that an autophagic response was implicated in the alkaline toxicity. In summary, the toxic response of alkali in *E. sinensis* was associated with oxidative stress, lipid metabolism disruption, apoptosis, and autophagy.

## Electronic supplementary material

Below is the link to the electronic supplementary material.

Supplementary Material 1

## Data Availability

The datasets generated and analysed during the current study are not publicly available due [The project is not yet completed] but are available from the corresponding author on reasonable request.
