# Supplementary material for: Effects of alkali stress on antioxidant capacity, lipid metabolism, apoptosis and autophagy of Eriocheir sinensis
Source: Sci Rep. 2025 Jul 1;15:22224. doi: 10.1038/s41598-025-96808-8 (PMC12217390; doi:10.1038/s41598-025-96808-8)
Supplement: Supplementary file 1 — Supplementary Material 1 [file 41598_2025_96808_MOESM1_ESM.docx]

**Supplementary materials**

**
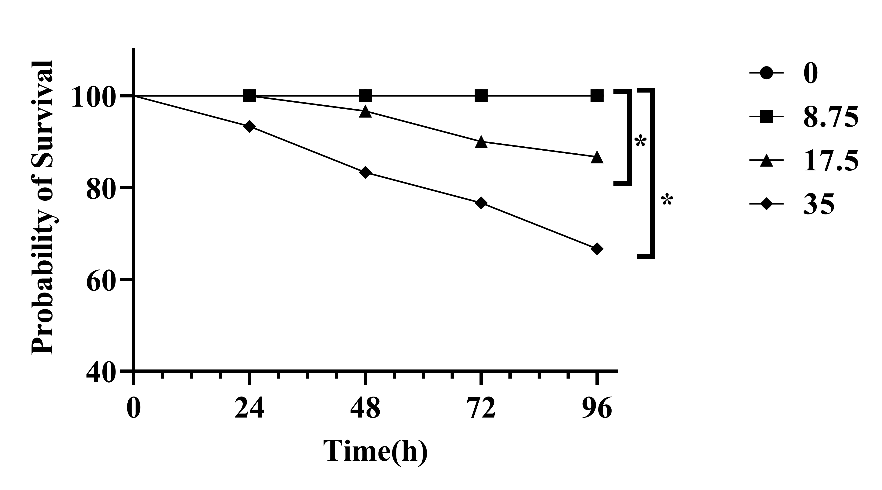
**As shown in Fig.S1, compared with 0 mmol/L group, the survival rate of *Eriocheir sinensis* in 17.5 mmol/L and 35 mmol/L alkalinity group significantly decreased (*P*<0.05).

Fig.S1 Survival rate of *Eriocheir sinensis* exposed to different concentrations alkalinity.

Note：Asterisks indicate significant differences between two groups (*P*<0.05).

The hepatopancreas structure of *Eriocheir sinensis* under alkali stress is shown in Fig.S2, the damage degree of the hepatopancreas increased gradually with increasing stress. In 17.5 and 35mmol/L group, the hepatic tubule lumen was dilatation, and a large amount of exfoliated basal membrane. Besides, in 35 mmol/L, the vacuoles were increased.

**
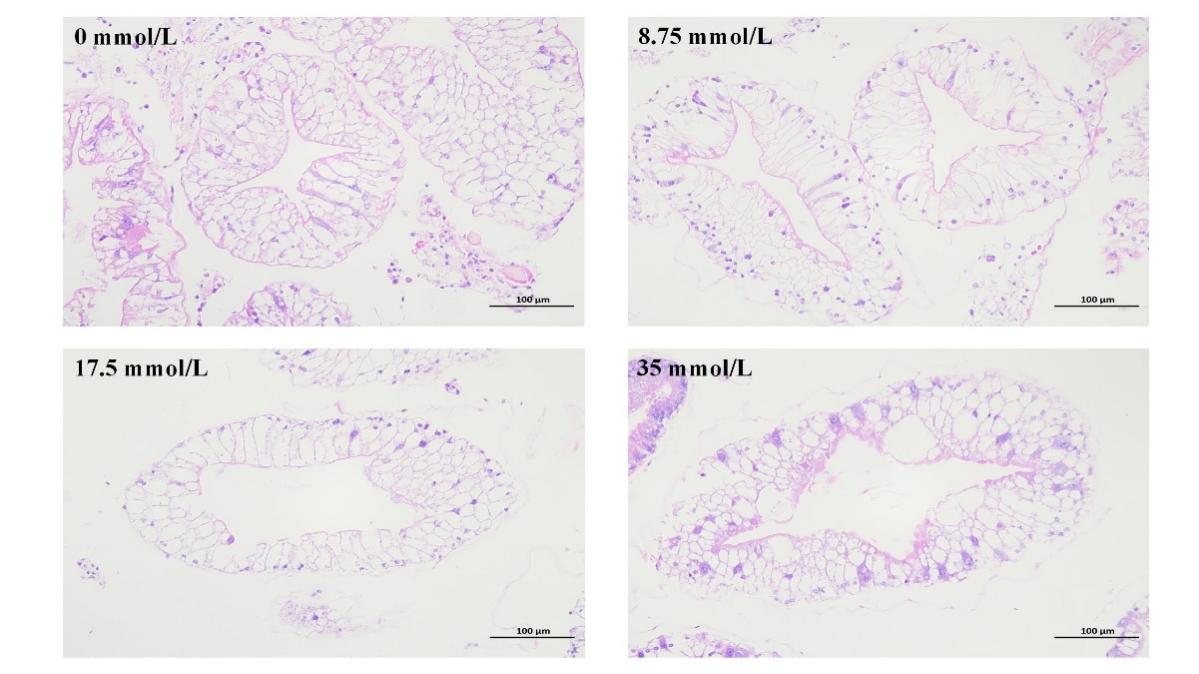
**

Fig.S2 Histological section of the hepatopancreas of *Eriocheir sinensis* under alkali stress.

Note：Asterisks indicate significant differences between two groups (*P*<0.05).

The results for ROS (Fig.S3) indicated that adding 17.5 and 35 mmol/L of alkalinity significantly increased ROS levels in the hepatopancreas (*P*<0.05).

**A:**

**
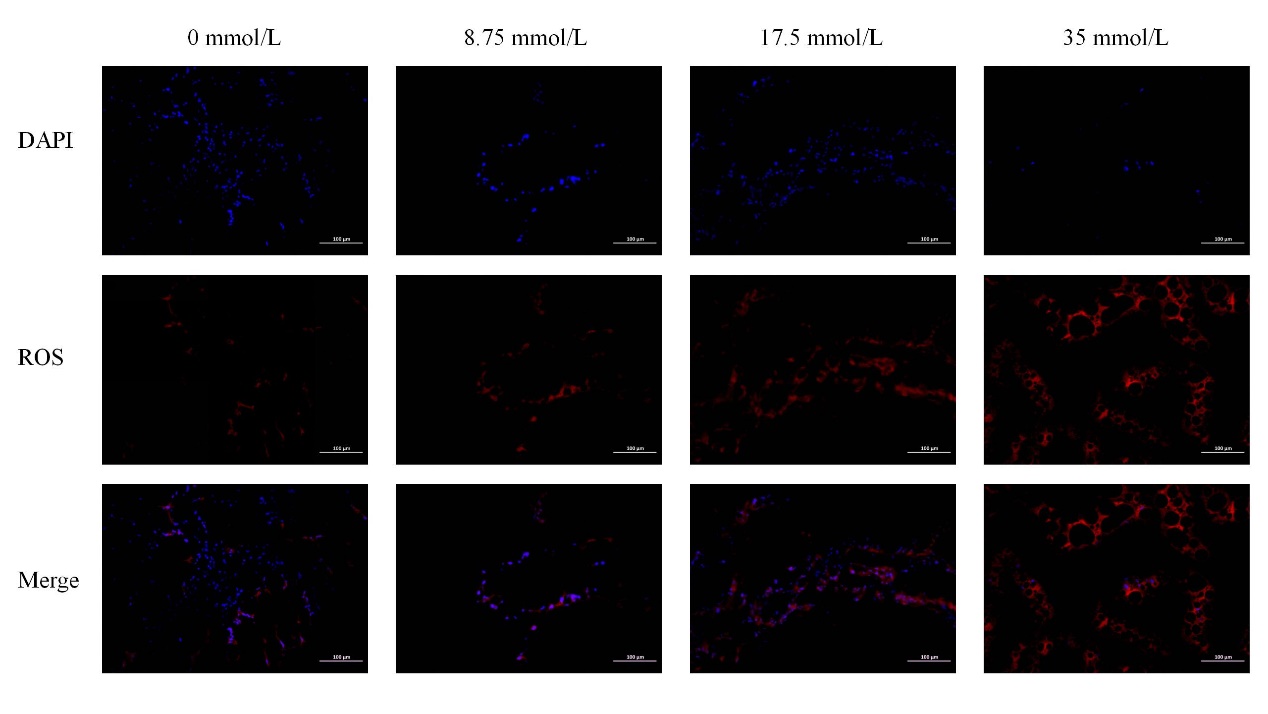
**

**B:**

**
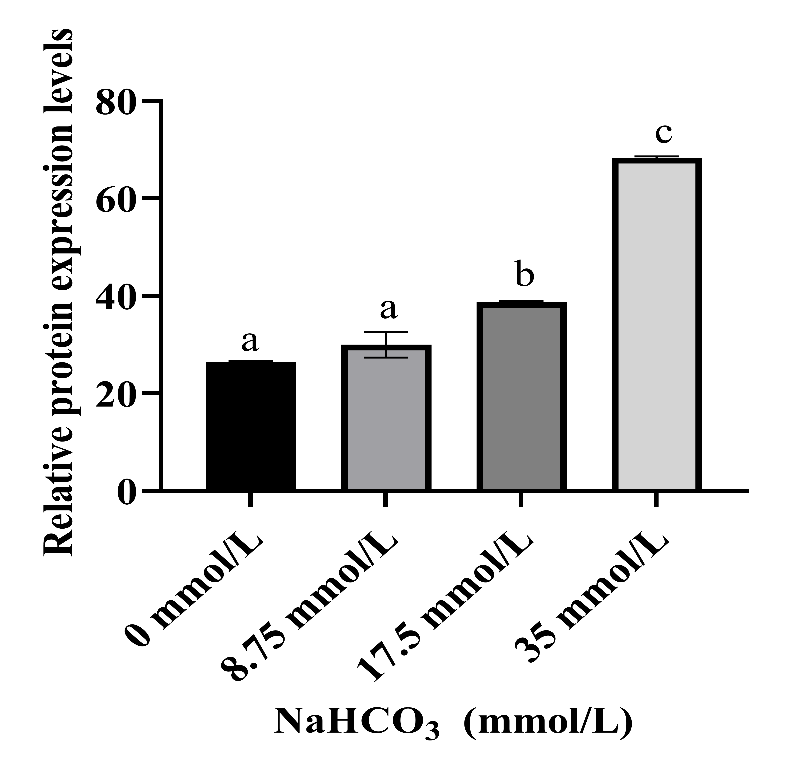
**

Fig.S3 The influence of alkalinity exposure on reactive oxygen species levels in hepatopancreas of *Eriocheir sinensis*

Note：Asterisks indicate significant differences between two groups (*P*<0.05).

Based on the above results, 17.5 mmol/L was selected as the experimental concentration.


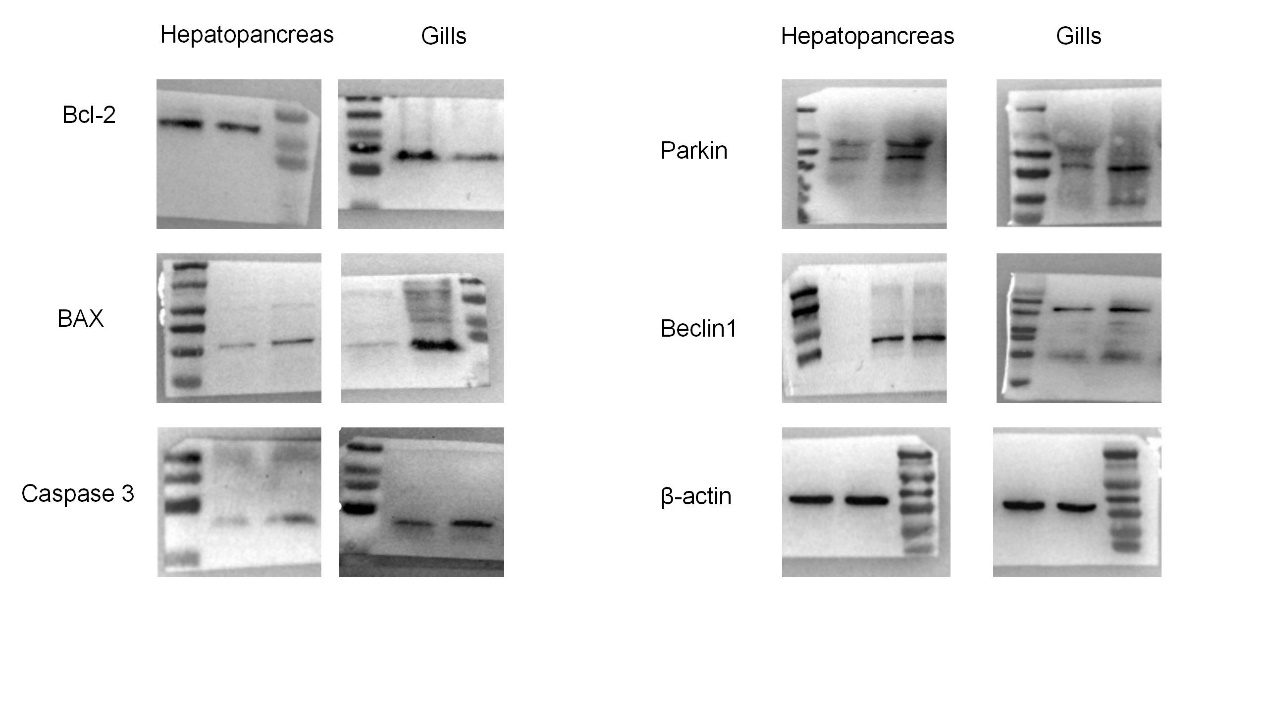


**Fig.S4** The original image of the protein

**Table S1:** Water quality parameters in this study

| Groups | pH | Temperature (℃) | DOC (mg/L) | salinity (ppm) |
| --- | --- | --- | --- | --- |
| 0 | 7.47±0.02 | 23.5±0.06 | 7.42±0.014 | 272.6±0.9 |
| 8.75 | 8.10±0.09 | 23.4±0.03 | 7.37±0.021 | 614.7±1.5 |
| 17.5 | 8.21±0.06 | 23.5±0.07 | 7.41±0.019 | 849.7±3.7 |
| 35 | 8.50±0.02 | 23.4±0.07 | 7.40±0.010 | 1580.0±5.8 |

**Table S2: The sequences of genes**

| **Type** | **Genes** | **Primer sequence (5’-3’)** | **Amplicon size (bp)** | **Tm** | **Accession number** | **Reference** |
| --- | --- | --- | --- | --- | --- | --- |
| Internal reference | *β-actin-*F | CAGGAAATGACCACTGCCGC | 95 | 61.9 | KY356885.1 | (Zeng, et al., 2024) |
|  | *β-actin-*R | CGGAACCTCTCATTGCCGA |  | 59.7 |  |  |
| Apoptosis | *Caspase3*-F | AGGAAAAGTTCACGCCGCTA | 103 | 59.9 | MH183147.1 | (Zheng, et al., 2022) |
|  | *Caspase3*-R | GGCTGCCTTCTGTCAGGATT |  | 60.0 |  |  |
|  | *Bcl2*-F | GCTCAGGGCAGCGTGT | 493 | 59.6 | [XM_050860189.1](https://www.ncbi.nlm.nih.gov/entrez/viewer.fcgi?db=nucleotide&id=2311898765) | (Bu, et al., 2022) |
|  | *Bcl2*-R | GCAACCCAGACTCAATCAA |  | 55.4 |  |  |
|  | *p53*-F | ATGTGCCTTGGCTCCAGTGTTG | 149 | 63.6 | [XM_050835363.1](https://www.ncbi.nlm.nih.gov/entrez/viewer.fcgi?db=nucleotide&id=2311988910) | (Bu, et al., 2022) |
|  | *p53*-R | TCGTCAGTCTTGATGTCTCGTGTG |  | 62.6 |  |  |
|  | *Bax*-F | AGAGATGAAGCAGACCACGC | 106 | 60.1 | c143681_g1 | (Zheng, et al., 2022) |
|  | *Bax*-R | TTCTACGGTGGGTGAGTCCA |  | 60.1 |  |  |
| Antioxidant | *Keap1-*F | CAACACCTTCATTGAGCAGCAC | 106 | 60.6 | MW818450.1 | (Zeng, et al., 2024) |
|  | *Keap1-*R | CATTGTACACGTCCTTCTCGTCT |  | 60.3 |  |  |
|  | *SOD*-F | GATGAAGCGCGTGTGATTCGT | 118 | 61.9 | FJ617306.1 | (Zheng, et al., 2022) |
|  | *SOD*-R | TATGGCTAAACATCGCCGCA |  | 60.1 |  |  |
|  | *CAT*-F | ATCCTGCTGCAGGACATCCAA | 127 | 61.8 | GU 361618.1 | (Zheng, et al., 2022) |
|  | *CAT*-R | TGATGTCGTGGGTGACCTCAAA |  | 61.8 |  |  |
|  | *Nrf2*-F | GCATCCTTCTGGTACCTCGTT | 91 | 59.7 | MW818449.1 | (Zeng, et al., 2024) |
|  | *Nrf2*-R | CACTGCTTTGGCTCATCCTTG |  | 59.8 |  |  |
|  | *HSP70*-F | GGCAAGGCAGCGAAGGTCATC | 127 | 64.2 | [XM_050870185.1](https://www.ncbi.nlm.nih.gov/entrez/viewer.fcgi?db=nucleotide&id=2311930944) | (Zhang, et al., 2023) |
|  | *HSP70*-R | CGGCATTGGTGACAGACTGACG |  | 63.9 |  |  |
|  | *HSP90*-F | TCACCAACGACTGGGAGGAT | 83 | 60.5 | [XM_050873094.1](https://www.ncbi.nlm.nih.gov/entrez/viewer.fcgi?db=nucleotide&id=2311938251) | (Zhang, et al., 2023) |
|  | *HSP90*-R | CAGGAAGAGGAGTGCCCTGA |  | 60.9 |  |  |
| Autophagy | *Atg5*-F | ACCAGCAGGACGCAGAGATGT | 134 | 63.8 | [XM_050849121.1](https://www.ncbi.nlm.nih.gov/entrez/viewer.fcgi?db=nucleotide&id=2311877130) | (Wang, et al., 2023) |
|  | *Atg5*-R | GTGTGAGAAGTGTGCCGTGAGG |  | 63.5 |  |  |
|  | *Atg7*-F | TCCGACTTCATCCGAAAATACC | 35 | 58.2 | MT543027.1 | (Feng, et al., 2022) |
|  | *Atg7*-R | GCACTCAACCCCAAGCCTG |  | 61.2 |  |  |
|  | *Beclin1*-F | GCCCATATACTGTGGCGAGG | 176 | 60.3 | MH173046.1 | (Feng, et al., 2022) |
|  | *Beclin1*-R | CCAGGTCAAAGAGCCCAGTT |  | 59.8 |  |  |
|  | *Lc3a*-F | ACGTCACGATGGGAGAACTG | 140 | 59.7 | [XM_050842840.1](https://www.ncbi.nlm.nih.gov/entrez/viewer.fcgi?db=nucleotide&id=2312014437) | (Feng, et al., 2022) |
|  | *Lc3a*-R | GTGGTGGTGCTCGTAAACCT |  | 60.2 |  |  |
| Lipid metabolism | *FAS*-F | GTCCCTTCTTCTACGCCATCC | 127 | 60.3 | - | (Lin, et al., 2020) |
|  | *FAS*-R | CGCTCTCCAGGTCAATCTTCAC |  | 61.3 |  |  |
|  | *CPT-1-F* | CATCTGGACACCCACCTCCA | 183 | 60.8 | - | (Lin, et al., 2020) |
|  | *CPT-1-R* | ATCTCCTCACCCGGCACTCT |  | 60.7 |  |  |
|  | CPT-2-F | AGCAGGCAGTGGCTCAGTTTA | 169 | 62.0 | MH037160.1 | (Huang, et al., 2023) |
|  | CPT-2-F | AAGGCAAGGAAGGGGTTGTAG |  | 59.9 |  |  |
|  | *CAAT-F* | CATCAAGAGCCAGGAGCCCA | 172 | 63.2 | - | (Lin, et al., 2020) |
|  | *CAAT-R* | CTTCAACAGCAGCCCGCAAA |  | 64.5 |  |  |
|  | *SREBP-F* | AGGGCTTCCAGCACGAC | 189 | 59.26 | [XM_050880186.1](https://www.ncbi.nlm.nih.gov/entrez/viewer.fcgi?db=nucleotide&id=2311961721) | (Yang, et al., 2022) |
|  | *SREBP-R* | CTTTGCCACAGATAACAGACG |  | 57.27 |  |  |

Bu, X., Song, Y., Pan, J., Wang, X., Qin, C., Jia, Y., Du, Z., Qin, J.G., Chen, L., 2022. Toxicity of chronic copper exposure on Chinese mitten crab (Eriocheir sinensis) and mitigation of its adverse impact by myo-inositol. Aquaculture. 547, 737511-.<https://doi.org/10.1016/j.aquaculture.2021.737511>

Feng, W., Su, S., Song, C., Yu, F., Zhou, J., Li, J., Jia, R., Xu, P., Tang, Y., 2022. Effects of Copper Exposure on Oxidative Stress, Apoptosis,Endoplasmic Reticulum Stress, Autophagy and Immune Response in Different Tissues of Chinese Mitten Crab (*Eriocheir sinensis*). antioxidants. 11, 2029.<https://doi.org/10.3390/antiox11102029>

Huang, Q., Wang, X., Lin, Z., Liu, J., Wang, H., Zhang, C., Du, Z., Qin, J., Chen, L., 2023. Regulatory role of vitamin A in lipid metabolism and health of Eriocheir sinensis fed different vegetable oil sources. Aquaculture. 568, 739324.<https://doi.org/10.1016/j.aquaculture.2023.739324>

Lin, Z., Han, F., Lu, J., Guo, J., Qi, C., Wang, C., Xiao, S., Bu, X., Wang, X., Qin, J., Chen, L., 2020. Influence of dietary phospholipid on growth performance, body composition, antioxidant capacity and lipid metabolism of Chinese mitten crab, Eriocheir sinensis. Aquaculture. 516.<https://doi.org/10.1016/j.aquaculture.2019.734653>

Wang, J., Sun, L.M., Li, X., Tao, S., Wang, F., Shi, Y., Guan, H., Yang, Y., Zhao, Z., 2023. Alkali exposure induces autophagy through activation of the MAPKpathway by ROS and inhibition of mTOR in Eriocheir sinensis. Aquatic toxicology. 258, 106481.<https://doi.org/10.1016/j.aquatox.2023.106481>

Yang, Z., Lian, W., Waiho, K., Zhu, L., Chen, A., Cheng, Y., Wang, Y., 2022. Effects of copper exposure on lipid metabolism and SREBP pathway in the Chinese mitten crab *Eriocheir sinensis*. chemosphere. 308, 136556.<https://doi.org/10.1016/j.chemosphere.2022.136556>

Zeng, L., Wang, Y.H., Ai, C.X., Zhang, B., Zhang, H., Liu, Z.M., Yu, M.H., Hu, B., 2024. Differential effects of oxytetracycline on detoxification and antioxidant defense in the hepatopancreas and intestine of Chinese mitten crab under cadmium stress. Science of the Total Environment. 930.<https://doi.org/10.1016/j.scitotenv.2024.172633>

Zhang, R., Zhao, Z., Li, M., Luo, L., Wang, S., Guo, K., Xu, W., 2023. Effects of saline-alkali stress on the tissue structure, antioxidation, immunocompetence and metabolomics of *Eriocheir sinensis*. Science of the Total Environment. 871, 162109.<https://doi.org/10.1016/j.scitotenv.2023.162109>

Zheng, N., Wang, N., Wang, Z.Y., Abdallah, G., Zhang, B.Y., Wang, S., Yao, Q., Chen, Y.K., Wang, Q.J., Zhang, D.M., 2022. Effect of infection with Aeromonas hydrophila on antioxidant capacity, inflammation response, and apoptosis proteins in Chinese mitten crab (Eriocheir sinensis). Comparative Biochemistry and Physiology Part C: Toxicology & Pharmacology. 252, 109220-.<https://doi.org/10.1016/j.cbpc.2021.109220>

**Table S3: The the relative fluorescence intensity values for the TUNEL**

| Hepatopancreas  (0 mmol/L) | Hepatopancreas  (17.5 mmol/L) | Gills  (0 mmol/L) | Gills  (17.5 mmol/L) |
| --- | --- | --- | --- |
| 1.00±0.01 | 3.10±0.03 | 1.01±0.08 | 1.34±0.037 |

**Table S4: The Mean ± SEM values for the autophagy genes**

| Genes | Hepatopancreas  (0 mmol/L) | Hepatopancreas  (17.5 mmol/L) | Gills  (0 mmol/L) | Gills  (17.5 mmol/L) |
| --- | --- | --- | --- | --- |
| ATG5 | 1.01±0.11 | 4.43±0.90 | 1.02±0.14 | 2.08±0.36 |
| Beclin1 | 0.98±0.04 | 8.51±0.80 | 1.07±0.27 | 4.08±0.22 |
| ATG7 | 1.00±0.06 | 6.92±0.03 | 1.04±0.20 | 5.54±0.23 |
| LC3a | 1.01±0.11 | 3.44±0.27 | 1.02±0.14 | 6.02±0.28 |
